# Perceptions and Practices of Healthcare Professionals in Managing Pediatric Obesity: Insight from a Focus Group Interviews in South Korea

**DOI:** 10.3390/children13060760

**Published:** 2026-05-29

**Authors:** Yoon Lee, Minsoo Shin, Jahye Jung, Ah-Ram Sul, Yong Hee Hong

**Affiliations:** 1 Department of Pediatrics, Korea University Medical Center Anam Hospital, Seoul 02841, Republic of Korea; ragabash@korea.ac.kr; 2 Department of Pediatrics, Korea University Ansan Hospital, Ansan 15355, Republic of Korea; sms1226@kumc.or.kr; 3 Division of Health Technology Assessment Research, National Evidence-Based Healthcare Collaborating Agency, Seoul 04933, Republic of Korea; freesia416@neca.re.kr; 4 Department of Pediatrics, Soonchunhyang University Bucheon Hospital, Soonchunhyang University College of Medicine, Bucheon 14584, Republic of Korea

**Keywords:** pediatric obesity, overweight, healthcare professional, focus groups, qualitative research, Korea

## Abstract

**Highlights:**

**What are the main findings?**

- Healthcare professionals in South Korea identified multiple barriers to pediatric obesity management, including lack of multidisciplinary resources, low patient and family awareness, high dropout rates, and in-sufficient reimbursement.
- Most clinicians were unaware of existing governmental programs for pediatric obesity, revealing a critical gap between policy and clinical practice.

**What is the implication of the main finding?**

- Effective pediatric obesity care requires stronger multidisciplinary collaboration, better integration of community and school-based programs, and adequate reimbursement support.
- These findings may inform other countries with similar healthcare systems about context-specific barriers and the need for tailored, policy-linked approaches.

**Abstract:**

Background/Objectives: Pediatric obesity poses significant public health challenges and is associated with an increased risk of adult obesity. Healthcare professionals play an important role in providing patient-centered care; however, barriers to effective pediatric obesity management remain insufficiently explored in South Korea. Methods: Eleven healthcare professionals managing pediatric obesity participated in focus group interviews. Audio-recorded interviews were transcribed verbatim and analyzed using thematic content analysis. Four main themes emerged: (1) the current status of pediatric obesity management, (2) clinical experiences and outcomes, (3) awareness of governmental policies, and (4) areas needing improvement. Results: Participants described multiple barriers to pediatric obesity management, including limited clinical resources, time constraints, low awareness among patients and families, and perceived inadequacies in reimbursement policies. Participants also reported low treatment adherence and frequent dropout during follow-up care. Many participants reported limited awareness of governmental initiatives related to pediatric obesity management but expressed willingness to utilize such programs if they became more accessible and better integrated into clinical practice. Conclusion: The findings suggest that pediatric obesity management in South Korea may be influenced by structural, financial, and sociocultural barriers. Participants emphasized the need for improved multidisciplinary collaboration, greater institutional support, and increased accessibility of obesity-related programs and resources. Further comparative and context-specific studies may help inform tailored approaches to pediatric obesity management.

## 1. Introduction

Pediatric obesity is a significant public health concern that leads to adult obesity. It presents challenges for effective treatment and underscores the need for lifelong healthy eating and lifestyle habits [1,2]. Additionally, it imposes substantial economic costs and elevates the risk of health complications, including type 2 diabetes and dyslipidemia [3,4,5]. In Korean adolescents, obesity is also associated with increased risks of hypertension and metabolic syndrome, further emphasizing the need for early intervention [6]. According to the Korea National Health and Nutrition Examination Survey conducted from 2019 to 2022, the prevalence of overweight children and adolescents was 23.5%, obesity was 14.2%, Class I obesity was 2.5%, and Class II obesity was 0.5% [7]. Among East Asian countries, South Korea has experienced a notable increase in pediatric obesity, as reflected in both regional comparative analyses and recent national surveillance data [8,9]. For effective treatment of obesity, a multidisciplinary therapeutic approach is essential [10,11,12]. A comprehensive, patient-centered family treatment program can boost engagement and adherence. Achieving this level of lifestyle modification requires close collaboration among all specialists involved, including physicians, dietitians, psychologists, social workers, and exercise specialists [13]. Although parents play a vital role in the early identification and intervention of childhood obesity and in shaping children’s health behaviors, a coordinating healthcare professional can assume a central role in successful patient-centered care [14,15,16].

South Korea is one of the most well-organized countries in terms of its National Health Insurance System (NHIS), a compulsory health insurance scheme covering the entire Korean population [17]. However, NHIS in Korea currently offers no coverage for obesity prevention, counselling or pharmacological treatment for obesity. The only obesity-related intervention covered by the Korean NHIS is bariatric surgery, which has been reimbursed since 2019. Even then, it is limited to morbidly obese patients with a body mass index (BMI) ≥ 35 kg/m^2^ or ≥30 kg/m^2^ in the presence of comorbidities, such as hypertension or diabetes—leaving a substantial gap in coverage for pediatric obesity management.

Given the rising prevalence of pediatric obesity in Korea and the limitations in existing healthcare policies, it is important to better understand the real-world challenges of pediatric obesity management. Qualitative studies in Western primary care settings have identified several recurring barriers to addressing childhood obesity, including clinicians’ reluctance to initiate weight-related conversations, fear of stigmatizing patients or damaging therapeutic relationships, and ambiguity around professional accountability [18,19,20,21]. Similar challenges have also been reported in South American contexts [22,23]. However, limited qualitative research has examined how healthcare professionals perceive pediatric obesity management within the South Korean healthcare system, where clinical practice patterns, reimbursement structures, and sociocultural perceptions of childhood obesity may differ substantially from those reported in other countries. Building on findings from our previous nationwide physician survey [24], which identified substantial barriers to pediatric obesity management in Korea, this qualitative study aimed to explore healthcare professionals’ experiences and perspectives on pediatric obesity management, including their clinical challenges, perceptions of current health policies, and suggestions for systemic improvement. To our knowledge, this is the first qualitative study to examine healthcare professionals’ perspectives on pediatric obesity management within the South Korean healthcare system.

## 2. Materials and Methods

### 2.1. Ethical Considerations

This study was approved by the National Evidence-based Healthcare Collaborating Agency Institutional Review Board in Seoul, Republic of Korea (Approval Number: NECAIRB24-006). All participants provided informed consent prior to participation.

### 2.2. Study Design

**Figure 1:**
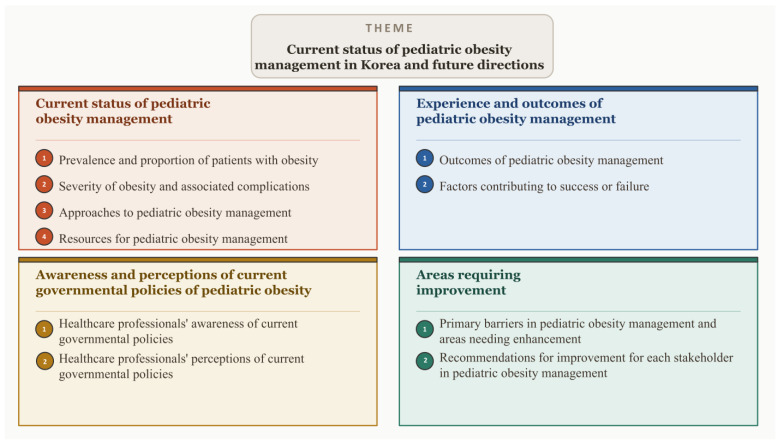
Summary of themes discussed in focus group discussions and interviews.

Focus group interviews (FGIs) represent a qualitative research methodology that elicits comprehensive data through structured discussions on specific topics. Each FGI involved 5–6 participants and provided detailed insights and personal expressions often overlooked in quantitative surveys. This method facilitates participant interaction, leading to new ideas and reflections that may not emerge in individual interviews [25]. The questions of this FGIs were based on a semi-structured interview protocol that included inquiries on: (1) current status of pediatric obesity treatment (four questions), (2) experiences with pediatric obesity treatment in clinical practice (two questions), (3) opinions on existing national health policies (four questions), and (4) feedback on areas requiring improvement (three questions) (Figure 1). The interviews were conducted by the research firm Hankook research (Seoul, Republic of Korea).

### 2.3. Participants and Recruitment

This study included healthcare professionals actively engaged in the management of pediatric obesity. To ensure representation from key experts in the field, information about the study was disseminated through online channels of the Korean Pediatric Society, the Korean Society for the Study of Obesity, the Korean Society of Pediatric Endocrinology and the Korean Society of Pediatric Gastroenterology, Hepatology and Nutrition, which are the leading academic organizations representing pediatric obesity specialists in Korea. These organizations serve as the primary networks for healthcare professionals dedicated to advancing knowledge and practice in pediatric obesity care.

A total of eleven participants were recruited from among the members of these societies. The characteristics of the participants, including their affiliated centers, years of clinical practice, specialized fields, and the geographic locations of their centers, are summarized in Table 1.

### 2.4. Data Collection

Face-to-face interviews were conducted in two sessions on 18 June 2024 and 25 June 2024, moderated by two authors (Hong YH and Lee Y) who are experienced medical professionals in pediatric obesity management. Each session involved a group of five and six participants, with each interview lasting approximately two hours. All interviews were audio recorded and transcribed for analysis. Before the FGI sessions began, participants were informed about the research objectives, methods, confidentiality of the interview contents, and their right to withdraw. Participants took part in the FGIs after providing written consent. Additionally, they were informed that they could discuss any difficulties or choose to stop the interviews at any time. The moderators had no prior personal or supervisory relationships with any of the participants. The moderators’ clinical expertise in pediatric obesity informed the design of the interview protocol and facilitated in-depth exploration of clinical experiences; however, this was recognized as a potential source of interpretive bias, which was mitigated through the use of a structured protocol administered by Hankook Research and through collaborative analysis and reflexive discussion among the research team. The FGI process and research steps are summarized in Table 2.

### 2.5. Data Analysis

Transcripts were prepared and initially coded by Hankook Research. The interviews were then analyzed using qualitative content analysis to identify textual differences and similarities, following the framework established by Braun and Clarke [26,27]. Three authors (Hong YH, Shin M, and Lee Y) independently reviewed all transcripts and the initial coding framework multiple times, refined and verified the codes, and extracted relevant meaning units, condensing them without losing their core meaning. The data were then entered into a computer template to ensure transparency. Discrepancies in interpretation were resolved through iterative discussion until consensus was reached. Thematic saturation was assessed iteratively; no new themes emerged in the second FGI session, indicating adequate informational sufficiency for the scope of this study. An audit trail was maintained throughout the analytical process to enhance trustworthiness. The study design, data collection, and reporting followed COREQ guidelines. No dedicated qualitative data analysis software was used; analysis was conducted using a structured manual template and audit trail to ensure transparency.

## 3. Results

The main themes and sub-themes identified from the focus group interviews are summarized in Table 3.

### 3.1. Current Status of Pediatric Obesity Management

#### 3.1.1. Prevalence and Proportion of Patients with Obesity

Affiliated centers exhibit significant variations in the number of children with obesity and their characteristics. Participants from primary clinics reported encountering a higher proportion of children with obesity compared to those in larger centers, while also noting lower levels of obesity awareness among patients and caregivers in these settings.

*“Based on my observation, I see 5 to 8 follow-up patients each week, which amounts to approximately 25 to 35 patients per month when including first visits.” (ID #1, Pediatric gastroenterologist/teaching hospital)*

*“I believe the situation differs significantly between the teaching hospitals and primary clinics. Can you estimate the number of obese children among one hundred visits to a primary clinic? It is quite substantial, yet none of them seem to recognize their obesity or express a desire to be assessed for it. Nearly one-third of children I encounter are obese, but they typically visit for simple upper respiratory infection care. As a result, a large number of obese children are being neglected.” (ID #4, General pediatrics/primary clinic)*

#### 3.1.2. Severity of Obesity and Associated Complications

Participants from teaching hospitals reported seeing more cases of morbid obesity, while those in primary clinics noted that early-stage metabolic complications were also commonly encountered.

*“I believe it is more common for patients to visit due to abnormal laboratory results rather than simply for obesity.” (ID #1, Pediatric gastroenterologist/teaching hospital)*

*“For me, visits from children with simple obesity are rare; most of my pediatric patients experience developmental delays, and many of them also have obesity, primarily severe obesity. In fact, obesity in developmental delay children is associated with parental factors, including the inability to provide proper care for the child.” (ID #11, Rehabilitation medicine specialist/teaching hospital)*

*“It is a considerable number. Children with elevated liver function tests or dyslipidemia, though not necessarily indicating the need for pharmacotherapy. I estimate that this accounts for about 30 to 40 percent.” (ID #6, Pediatric endocrinologist/primary clinic)*

#### 3.1.3. Approaches to Obesity Management

Participants commonly described nutritional counseling and lifestyle modification education as their primary management strategies, but noted that a multidisciplinary or structured approach was difficult to implement due to constraints in time, workforce, and equipment. Most centers lack other professionals such as dietitians and exercise specialists; therefore, physicians are responsible for all counselling and education. Exercise therapies are typically provided through educational materials and parental education, but compliance and outcomes are often unsatisfactory. In many cases, the effects of pharmacotherapy are insufficient, primarily due to a lack of accompanying lifestyle modifications.

*“Recommending aggressive treatment for obesity early on is a challenging decision. Therefore, the mainstay of my approach is education. We also provide dietary counselling; however, as you may be aware, we cannot bill for this service, which imposes certain limitations.” (ID #3, Pediatric gastroenterologist/teaching hospital)*

*“As we all know, the fundamental principle is education for both parents and patients. Following that, lifestyle changes should be the first priority. Then, we provide nutritional education on our own; however, we cannot bill for that service….” (ID #5, Pediatric endocrinologist/secondary hospital)*

*“Middle and high school students often lack time for exercise because they attend private institutions. These children come home late at night, around 10:30 to 11 PM, leaving them with no time for physical activity at all.” (ID #1, Pediatric gastroenterologist/teaching hospital)*

*“I have used pharmacotherapy in limited cases, primarily when no other options seemed viable. In my experience, approximately 60% to 70% of patients—around two-thirds—show improvement. However, a significant challenge is the frequent occurrence of weight regain.” (ID #3, Pediatric gastroenterologist/teaching hospital)*

#### 3.1.4. Resources for Obesity Management (Time, Workforce, Equipment, Educational Materials)

Most participants indicated that resources related to obesity management are lacking, making it difficult to allocate sufficient time for patient education. They also noted a shortage of financial reimbursement to retain multidisciplinary professionals across various fields of obesity management, as well as insufficient equipment. Additionally, they emphasized the need to develop educational materials that are appropriate for this clinical environment.

*“There are no supporting resources available at all. The individuals who measure children’s weight and height are not trained nurses or medical professionals; they are simple office workers. As for waist circumference, I measure it myself. Blood pressure is measured using an automated sphygmomanometer, and if the reading is abnormal, then I ask the nurse to measure it manually. There is no nutritionist specializing in pediatric obesity. There are also no exercise specialists available.” (ID #1, Pediatric gastroenterologist/teaching hospital)*

*“What is important in primary care is the ability to determine a suitable treatment option within 2 to 3 min and to explain it to both the patient and the parent in a concise manner. To achieve this, the development of effective tools is essential.” (ID #6, Pediatric endocrinologist/primary clinic)*

*“For educational materials, my colleagues and I have collaboratively created some resources, and I utilize those materials with nothing but passion, without any compensation.” (ID #9, General pediatrician/primary clinic)*

Although the limited sample size precludes formal comparison, participants’ experiences appeared to differ meaningfully by clinical setting and specialty, suggesting that a uniform approach to pediatric obesity management may not be appropriate across all contexts.

### 3.2. Experience and Outcomes of Pediatric Obesity Management

#### 3.2.1. Outcomes of Pediatric Obesity Management

Based on personal clinical impressions, participants estimated that treatment success rates were generally below 30%, though these figures reflect subjective appraisals rather than formally measured outcomes. They also found a significant dropout rate during follow-up.

*“From my experience, only about 20% to 30% of patients achieve successful outcomes. The majority do not return for follow-up visits, which suggests that they have not effectively managed their treatment. Regarding those who do not return, I cannot assume that they are doing well or adhering to the guidance provided. Instead, it is more probable that they are struggling with weight management, which is the reason for their absence.” (ID #1, Pediatric gastroenterologist/teaching hospital)*

*“The treatment success rate is comparable to that of other centers, estimated to be approximately one–third or less, around 20% to 30%.” (ID #3, Pediatric gastroenterologist/teaching hospital)*

*“Although I have often heard success stories, I can’t help but feel that most of my patients have not achieved their goals. While I believed I was making progress, the outcomes, particularly in terms of weight management, have not been as I hoped.” (ID #7, Pediatric endocrinologist/secondary hospital)*

*“I believe there are almost no successful cases. There was one child who managed to lose some weight some time ago, but the child did not follow up effectively. They return for vaccinations after a while, and while they may maintain their weight temporarily, they ultimately end up regaining it.” (ID #6, Pediatric endocrinologist/primary clinic)*

#### 3.2.2. Factors Contributing to Success or Failure in Pediatric Obesity Management

Participants indicated that younger patients with a better awareness of their condition tend to have more favorable outcomes. The main causes of failure included: ‘socioeconomic status (SES) and the family’s living environment,’ ‘parental involvement in care,’ ‘awareness of obesity,’ ‘lack of support and infrastructure for obesity treatment, including reimbursement,’ and ‘a societal atmosphere that places excessive emphasis on academic achievement and college entrance.’

*“The environment is not conducive at all. One parent of my patient even asked, ‘Hey, doctor, do you eat and exercise as you teach?’ The answer is no, I do not either! This is largely because both parents are working and cannot prepare meals for their children. It is unrealistic to expect them to provide high-quality meals and ensure that their kids do not skip breakfast, especially when the mother lacks the time to cook.” (ID #1, Pediatric gastroenterologist/teaching hospital)*

*“Everyone has mentioned similar points, indicating that patients who are aware of their condition are more likely to succeed. In the case of a child referred to the hospital due to obesity detected incidentally, without motivation or support, they tend to struggle.” (ID #2, Pediatric gastroenterologist/teaching hospital)*

*“Although I am seeing more patients with obesity than my junior staff, my outpatient income is even lower than theirs. This is because the reimbursement amount for each patient is fixed, regardless of the time spent on education. This reflects the current reality of obesity treatment.” (ID #8, Family medicine doctor/teaching hospital)*

*“Pediatric obesity is important, but it is not perceived as urgent by them. Unlikely acute illnesses that need immediate resolution, the lack of control over obesity may not seem to make a significant difference in the short term. I believe this is a key issue in obesity management.” (ID #10, General pediatrician/primary clinic)*

Across themes, participants described an interconnected cycle of systemic barriers. Inadequate reimbursement constrained consultation time, limiting the depth of patient education and counselling. This, combined with high dropout rates, contributed to poor long-term outcomes. These findings suggest that financial disincentives not only affect individual consultations but also undermine the continuity of care necessary for effective pediatric obesity management.

### 3.3. Awareness and Perceptions of Current Governmental Policies of Pediatric Obesity

#### 3.3.1. Healthcare Professionals’ Awareness of Current Governmental Policies

Before the FGI sessions, most participants reported being unaware of existing governmental policies and programs related to pediatric obesity. However, once informed, they expressed willingness to introduce relevant programs and affiliated organizations to their patients.

*“I was not even aware that such programs or policies existed. If these were promoted to hospitals and physicians treating pediatric patients with obesity, we could recommend our patients to participate in these training and programs. It’s unfortunate that we missed this opportunity.” (ID #1, Pediatric gastroenterologist/teaching hospital)*

It is notable that participants’ lack of awareness was distinct from their critical evaluations of those programs’ effectiveness. Several participants who had been previously unaware expressed willingness to engage with existing initiatives once informed, while others who had some prior knowledge questioned their practical implementability.

#### 3.3.2. Healthcare Professionals’ Perceptions of Current Governmental Policies

Among participants who were familiar with existing policies, or upon reflection following the FGI discussion, views were mixed. Some acknowledged that governmental recognition of pediatric obesity as a public health priority was a positive step. However, participants commonly noted that the perspectives of frontline healthcare professionals had not been adequately incorporated into policy design, and that implementation in real-world clinical, school, and community settings remained challenging.

*“Notably, I have never encountered this before. Upon examining the situation, it appears to be a misallocation of budgetary resources that fails to address more critical areas.” (ID #6, Pediatric endocrinologist/primary clinic)*

*“Even if policies are in place, in real-world situations, teachers often have valid reasons for being unable to implement them. For instance, when children are outside playing, there may not be enough teachers available to supervise and if an accident occurs, the responsibility falls on the teacher. This creates challenges for schools in promoting outdoor activities. Ultimately, additional funding would be necessary—for example, to hire assistant teachers who can supervise students during outdoor activities.” (ID #10, General pediatrician/primary clinic)*

### 3.4. Areas Requiring Improvement

#### 3.4.1. Primary Barriers in Pediatric Obesity Management and Areas Needing Enhancement

The most frequently mentioned barrier to obesity treatment and area requiring improvement was ‘medical reimbursement rates.’ For hospitals to actively provide treatment and collaborate with other healthcare or social institutions, appropriate reimbursement levels must be established.

*“I believe the biggest barrier and the area that ultimately needs improvement is the reimbursement rates. If this issue is not addressed, education will not take place, and participation in educational programs will be insufficient. Consequently, the number of obese children will continue to rise, creating a vicious cycle.” (ID #6, Pediatric endocrinologist/* *primary clinic)*

#### 3.4.2. Recommendations for Improvement for Each Stakeholder in Pediatric Obesity Management

Regarding the most effective timing for obesity intervention, the healthcare professionals interviewed unanimously agreed that earlier intervention yields better results. One participant noted that developing healthy eating habits is essential, and schools are the most effective in situations for achieving this. Since it is hard for clinics to provide exercise therapy independently, establishing policies to connect with relevant local organizations and promoting these initiatives could support healthcare professionals in introducing such programs to children. Additionally, they emphasized the need to raise general awareness about obesity throughout society.

*“If having an exercise specialist is not feasible in a clinic, how about connecting with sports facilities where doctors can provide exercise prescriptions? This could allow patients to visit specialized centers to learn and view instructional videos. Establishing such connections would be highly beneficial.” (ID #1, Pediatric gastroenterologist/* *teaching hospital)*

*“Although children eat lunch together at school, the real issue lies with the dinner and snacks they have at home. For these children, it might be beneficial to provide dinner for those with obesity who require intensive management, possibly by collaborating with one or two schools to offer dinner or providing packed meals that they can take home. For children from low-income or single-parent families, where mothers may struggle to prepare nutritious meals and often resort to instant food, having dinner services available at school could make a significant difference.” (ID #9, General pediatrician/* *primary clinic)*

## 4. Discussion

Current findings from the FGI allow us to delineate the status of pediatric obesity management in Korea, as well as identify various barriers and facilitators. Most patients with pediatric obesity do not seek medical attention solely for obesity; rather, they present with complications or are referred following school health check-up findings. Children with morbid obesity or comorbidities, such as diabetes or nonalcoholic fatty liver disease, are more frequently seen in teaching hospitals, which is reflective of the medical service delivery system in Korea.

Conversely, participants suggested that the number of children with obesity presenting to primary and secondary healthcare centers appeared to be increasing, participants perceived that patients and parents in these settings showed lower awareness of obesity compared to those seen in teaching hospitals. This decline in awareness is concerning, as poor insight into obesity may be related to lower adherence to treatment and disease progression [28]. Therefore, effective obesity management in primary and secondary care settings is not only challenging but also critical for public health. And this discrepancy underscores the need for targeted education and interventions at the community level, particularly in primary care settings where early-stage obesity often goes unrecognized or unmanaged. The observed disparity between care levels reflects Korea’s tiered referral system, where complex cases concentrate in teaching hospitals. Primary care encounters earlier-stage obesity but with comparatively fewer resources and lower patient awareness—the very setting where timely intervention may matter most. Future policy should prioritize primary care capacity building alongside clearer referral pathways.

As most guidelines recommend, pediatric obesity management primarily involves nutritional counseling, lifestyle education, and exercise consultation [13,29,30]. However, there is a significant deficiency in resources allocated to obesity management, which makes it challenging to dedicate sufficient time to patient education. Additionally, the lack of financial reimbursement hampers the establishment of multidisciplinary teams of professionals across various domains of obesity management, compounded by inadequate equipment in all types of medical facilities. This situation makes it difficult to provide the tailored multidisciplinary treatment necessary for effective obesity management [29,31].

Currently, there is a notable dropout rate in obesity management. Caution is warranted in interpreting these figures, as there is no clearly defined endpoint for obesity treatment and follow-up [10,13,29,32]. Nevertheless, the participants in the FGI expressed frustration with these outcomes. Participants noted that Korea’s emphasis on academic achievement and private after-school tutoring limited children’s opportunities for physical activity, a concern consistent with national data showing that over 65–83% of elementary and middle school students attend after-school institutions [33]. These clinician-perceived cultural factors may be important considerations in designing obesity interventions tailored to the Korean context. Addressing these cultural and educational factors is crucial in designing effective obesity prevention and intervention programs tailored to the unique context of each country.

In terms of facilitators and barriers to obesity control, several key factors have been identified. These include ‘SES and the family living environment,’ ‘parental care,’ ‘poor awareness of obesity,’ ‘lack of support and infrastructure for obesity treatment, including reimbursement,’ and ‘a societal atmosphere that places excessive emphasis on academic achievement and college entrance’. Notably, poor insight and awareness of obesity has been frequently cited as a significant barrier in pediatric obesity management in previous studies [18,19,34,35,36].

The younger the patient is and the greater the awareness of the condition, the more parental support appears to act as a facilitator. This trend aligns with previous reports, with the exception of two factors: ‘the societal atmosphere that places excessive emphasis on academic achievement and college entrance’ and ‘the lack of support and infrastructure for obesity treatment, including reimbursement [37,38,39,40,41]. These two issues may be interpreted as Korea-specific challenges that require careful consideration when formulating pediatric obesity control policies.

The barriers identified in this study closely mirror the key barriers highlighted in FGI conducted with school staff in Australia [36]. These barriers include busy schedules, a shortage of trained staff and funding, a lack of consistency in introducing and implementing school interventions, and insufficient motivation among students with obesity. A qualitative systematic review conducted in the UK explored patients’ views on lifestyle interventions for obesity treatment. The review highlighted that adolescents emphasized the need for support from experts, families, and peers. Although they initially expressed concerns and fears about participating, once engaged, they valued long-term support, enjoyed sports and physical activities, and were motivated by the desire to improve their body image and enhance social acceptance [35].

Awareness of major current policies and programs is surprisingly low. While it is encouraging that the government acknowledges the seriousness of pediatric obesity, it appears that the perspectives of healthcare professionals actively involved in treatment are not being adequately reflected in these initiatives. Therefore, to achieve optimal results, it is essential to effectively promote these policies effectively and integrate community and school programs with medical interventions.

Regarding areas in need of improvement, ‘medical reimbursement rates’ was the most frequently mentioned concern. Previous studies support this assertion, indicating that these concerns are well-founded [42,43]. Appropriate reimbursement levels must be established to enable hospitals to actively provide treatment and connect with other healthcare and social institutions. While inadequate reimbursement was the most consistently cited concern, other factors identified in this study—including parental care, family living environment, and socioeconomic status—also play an important role in treatment outcomes. Furthermore, Korea’s cultural emphasis on academic achievement may impose additional constraints on behavior change regardless of available resources. A comprehensive policy approach should therefore address these multifactorial barriers alongside financial incentives. There is widespread agreement that early intervention and treatment during the initial stages of pediatric obesity are more effective in long-term health issues [13,20,44].

### Study Limitations

This study has several limitations. The sample was limited to members of specific academic societies, which may not fully represent the perspectives of all healthcare professionals involved in pediatric obesity management, such as nutritionists, exercise specialists, and others. Although the study intentionally focused on physicians as the primary gatekeepers of pediatric obesity diagnosis and referral within the Korean NHIS, future research should incorporate a multidisciplinary range of professionals to provide a more comprehensive understanding. Additionally, participant self-selection may have skewed perspectives toward more engaged or concerned clinicians, potentially overrepresenting motivated viewpoints. The group dynamics inherent to focus group interviews also introduce the possibility that dominant voices shaped discussion, while less assertive participants may have withheld differing views; although the moderator employed structured prompts to encourage balanced participation, this limitation cannot be fully eliminated. Finally, the qualitative nature of the study and the relatively small sample size limit the generalizability of the findings, which are intended to illuminate experiential themes and inform future quantitative research rather than produce statistically representative conclusions.

## 5. Conclusions

This study highlights important gaps, as perceived by a selected group of physicians, between the growing need for pediatric obesity care and the current healthcare infrastructure in Korea. Participating clinicians identified limited reimbursement, insufficient multidisciplinary resources, and difficulties in sustaining long-term lifestyle interventions as major barriers to effective care. They emphasized the need for sustainable care models that extend beyond tertiary hospitals and strengthen linkages with primary care. However, as findings reflect the perspectives of a small, specialty-engaged sample, they should not be generalized to all healthcare professionals or settings in Korea. Nevertheless, these insights may inform other countries facing similar challenges in building more accessible and sustainable pediatric obesity care systems.

## Figures and Tables

**Table 1:** General characteristics of interviewed healthcare professionals.

ID	Affiliated Centers	Years of Practice	Fields of Specialization	Detailed Locations
#1	Teaching Hospital	11	Pediatric GI	Seoul
#2	Teaching Hospital	6	Pediatric GI	Gyeonggi
#3	Teaching Hospital	7	Pediatric GI	Seoul
#4	Primary Clinic	15	General Pediatrics	Seoul
#5	Secondary Hospital	12	Pediatric EN	Seoul
#6	Primary Clinic	15	Pediatric EN	Seoul
#7	Secondary Hospital	3	Pediatric EN	Seoul
#8	Teaching Hospital	10	Family Medicine	Seoul
#9	Primary Clinic	4	General Pediatrics	Seoul
#10	Primary Clinic	10	General Pediatrics	Seoul
#11	Teaching Hospital	7	Pediatric RM	Seoul

Pediatric EN = pediatric endocrinology, Pediatric GI = pediatric gastroenterology and nutrition, Pediatric RM = pediatric rehabilitation medicine.

**Table 2:** Research Steps: From Preparation to Completion.

Step	Details
Step 1 (Preparation)	Identify recruitment methods and data collection tools. Finalize the research topic. Select and recruit participants. Decide on the schedule, location, and duration. Prepare interview guidelines.
Step 2 (Introduction Phase)	Conduct introductions, present the purpose of the study, and facilitate rapport-building among participants. Check participants’ demographic and social information.
Step 3 (Orientation)	Explain the research objectives and main discussion topics. Provide an overview of the timeline and session details. Confirm participation and engage participants in the discussion.
Step 4 (Interaction Phase)	Conduct interviews related to the core topic, including open-ended discussions. Explore additional questions as needed, allowing participants to freely exchange opinions.
Step 5 (Final Phase)	Conclude the interviews. Summarize participant feedback and complete field notes. Organize and analyze audio recordings and transcripts. Draft and finalize the research report.

**Table 3:** Summary of focus group interview results.

Theme	Sub-Theme	Key Findings	Representative Quote
Theme 1 Current status of pediatric obesity management	Prevalence & patient characteristics	Larger centers see more severe obesity; primary clinics have higher prevalence but lower patient/caregiver awareness of obesity	*“Nearly one-third of children I encounter are obese, but they typically visit for simple upper respiratory infection care.” (ID #4)*
	Severity & complications	Morbid obesity more frequently reported by teaching hospital physicians; metabolic complications (dyslipidemia, elevated LFTs) estimated by one participant at 30–40% of patients, based on clinical observation	*“I estimate that [dyslipidemia/elevated LFTs] accounts for about 30 to 40 percent.” (ID #6)*
	Management approaches	Nutritional counseling and lifestyle education are mainstays; pharmacotherapy used selectively; multidisciplinary approach hampered by resource constraints	*“The mainstay of my approach is education … we also provide dietary counselling; however, we cannot bill for this service.” (ID #3)*
	Resources	Widespread shortage of dietitians, exercise specialists, and equipment; no financial reimbursement for counseling; physicians handle all education	*“There is no nutritionist specializing in pediatric obesity. There are also no exercise specialists available.” (ID #1)*
Theme 2 Experience and outcomes of pediatric obesity management	Outcomes	Treatment success rate subjectively estimated at ~20–30% based on participants’ personal clinical impressions; high dropout rate; weight regain common after pharmacotherapy	*“Only about 20% to 30% of patients achieve successful outcomes. The majority do not return for follow-up visits.” (ID #1)*
	Factors for success/failure	Facilitators: Younger age, patient awareness, parental support Barriers: Low SES, poor obesity awareness, academic pressure, lack of reimbursement	*“Patients who are aware of their condition are more likely to succeed.” (ID #2)*
Theme 3 Awareness & perceptions of governmental policies	Awareness	Most HCPs were unaware of existing governmental obesity programs before the FGI; expressed willingness to refer patients once informed	*“I was not even aware that such programs or policies existed … It’s unfortunate that we missed this opportunity.” (ID #1)*
	Perceptions	Positive recognition that government acknowledges pediatric obesity; concern that HCP perspectives are not reflected; policies deemed difficult to implement in real-world settings	*“Even if policies are in place, in real-world situations, teachers often have valid reasons for being unable to implement them.” (ID #10)*
Theme 4 Areas requiring improvement	Primary barriers	Insufficient reimbursement rates most commonly cited; followed by lack of multidisciplinary resources, low patient/family awareness, and fragmented community linkages	*“The biggest barrier … is the reimbursement rates. If this issue is not addressed, education will not take place.” (ID #6)*
	Recommendations	Earlier intervention; link clinics to local exercise facilities; school-based dinner/nutrition programs; raise societal obesity awareness; involve HCPs in policy design	*“How about connecting with sports facilities where doctors can provide exercise prescriptions?” (ID #1)*

FGI, focus group interview; HCP, healthcare professional; SES, socioeconomic status; LFT, liver function test.

## Data Availability

The data presented in this study are available from the corresponding authors upon reasonable request. Data are not publicly available due to privacy restrictions.
